# The natural course of cystic pancreatic neuroendocrine tumours in MEN1

**DOI:** 10.1530/EO-24-0050

**Published:** 2025-02-04

**Authors:** Eline N M van Vliembergen, Carolina R C Pieterman, Annenienke C van de Ven, Arthur J A T Braat, Gerlof D Valk, Joanne M de Laat

**Affiliations:** ^1^ Department of Endocrine Oncology, University Medical Centre Utrecht, Utrecht, The Netherlands; ^2^ Department of Endocrinology, Radboud University Medical Centre, Nijmegen, The Netherlands; ^3^ Department of Radiology and Nuclear Medicine, University Medical Centre Utrecht, Utrecht, The Netherlands

**Keywords:** pancreatic neuroendocrine tumour, multiple endocrine neoplasia type 1, cystic, natural course, histopathology

## Abstract

Pancreatic neuroendocrine tumours (PanNETs) significantly impact life expectancy in multiple endocrine neoplasia type 1 (MEN1). Both solid and cystic pancreatic lesions are observed in MEN1, yet limited research has been focused on cystic lesions in MEN1. While solid PanNETs are generally considered to have an indolent course, the natural course of cystic lesions remains unclear. This study aims to provide more insights into the natural course of cystic PanNETs in MEN1. Patients with MEN1 and radiologically suspected PanNETs, treated at UMC Utrecht and Radboudumc between 2010 and 2023, were included. In the first part, we examined the characteristics of patients with tumours visible on imaging scans. In the second part, we investigated outcomes of pathological examinations, following resection of these lesions. A total of 136 patients were included, comprising 60 men and 76 women. The median follow-up was 5.1 years. [^68^Ga]Ga-DOTATOC PET/CT scans showed that both cystic and solid lesions demonstrated [^68^Ga]Ga-DOTATOC PET/CT uptake. The median growth of cystic and solid PanNETs was similar. Pathological examination of 38 resected tumours confirmed that cystic lesions identified on imaging were indeed PanNETs. Cystic lesions had a median diameter of 26 mm at the time of resection, compared to 18 mm for solid lesions, with comparable Ki-67 indices and mitotic counts. We conclude that cystic and solid PanNETs in MEN1 patients appear to be morphological variations of the same entity, suggesting that similar management approaches should be considered.

## Introduction

Multiple endocrine neoplasia type 1 (MEN1) syndrome is an inherited disease characterized by the development of pituitary, parathyroid and pancreaticoduodenal tumours (Goudet *et al.* 2011, Sakurai *et al.* 2012, Thakker *et al.* 2012, de Laat *et al.* 2016). Pancreatic neuroendocrine tumours (PanNETs) occur in 50–70% of MEN1 patients and are the primary cause of reduced life expectancy in MEN1 (Goudet *et al.* 2010). PanNETs represent a heterogeneous group of tumours, presenting either as hormonally active tumours (producing gastrin, insulin, glucagon, somatostatin, vasoactive intestinal peptide or growth hormone-releasing hormone) or as non-functioning tumours.

In MEN1, multiple pancreatic lesions often occur with varying appearances. Cystic pancreatic lesions pose a diagnostic and therapeutic challenge, as it is unclear what their natural course is. While cystic PanNETs have been documented in studies of sporadic PanNETs, no studies have specifically described the natural course of cystic PanNETs in a population consisting of MEN1 patients. Routine screening in MEN1 facilitates the detection and monitoring of small tumours, allowing for the study of their natural history. In contrast, sporadic PanNETs are often diagnosed at an advanced stage, necessitating surgical intervention, which obscures their natural course. Therefore, this study aims to analyse the characteristics and natural course of cystic pancreatic lesions occurring in patients with MEN1.

## Methods

Patients with MEN1 who were treated from January 2010 to December 2023 at UMC Utrecht and Radboudumc were selected from the longitudinal Dutch MEN1 Study Group (DMSG) cohort. Detailed information on this longitudinal database has been described previously (de Laat *et al.* 2013, van Beek *et al.* 2018). Patients with pancreatic lesions suspected to be solid or cystic PanNETs based on radiological imaging (MRI or CT) were included. Specialized radiologists, experienced in pancreatic imaging for MEN1 patients, were involved in the radiological assessment. To meet diagnostic criteria, lesions had to be consistently identified on consecutive imaging scans. Electronic medical records were retrospectively reviewed and analysed.

MEN1 diagnosis was confirmed by either a germline MEN1 mutation or the presence of at least one major manifestation along with a first-degree relative diagnosed with MEN1.

Patients were categorized into three distinct groups: i) solid lesions, ii) cystic lesions and iii) both solid and cystic lesions. Patients were assigned to the group with solid lesions if only solid lesions were discernible in imaging scans. Conversely, patients were assigned into the group with cystic lesions when solely cystic lesions were identified. Patients with both solid and cystic lesions simultaneously present were assigned to the group with both solid and cystic lesions. Patients in whom imaging reports were not available were excluded from the study.

Lesion growth was calculated by the increase in size divided by the years of follow-up, focusing only on the largest lesion. In accordance with the guidelines, patients underwent imaging at intervals ranging from six months to two years, depending on the size and growth of their PanNETs. Patients were excluded from this analysis if the size of their lesion could not be accurately measured due to suboptimal imaging quality. Patients treated with somatostatin analogues (SSAs), peptide receptor radionuclide therapy (PRRT) or chemotherapy were monitored until the initiation of these treatments. Patients who were already undergoing treatment at the start of follow-up, or patients with only one imaging report available before treatment, were excluded from the growth analysis. The same criteria applied to surgery and radiotherapy; the follow-up period was terminated at the time of the last imaging scan before intervention, and patients were excluded if no repeated imaging was available before treatment.

Metastatic disease was based on either histopathological examination or the detection of suspected lesions on MRI or CT, confirmed through [^68^Ga]Ga-DOTATOC PET/CT scan. The suspected PanNETs and/or metastases were required to be present on at least two consecutive scans for confirmation, without subsequent absence of the lesions. Patients in whom the origin of the metastases was unclear due to the simultaneous presence of other lesions that could potentially have caused the metastases (e.g. duodenal NETs), and pathological examination did not provide clarification were excluded from this analysis.

In the second part of our study, we focused on the outcomes of pathological examinations following surgical resection of pancreatic lesions suspected to be PanNETs between January 2010 and December 2023. Patients were categorized into two groups: patients with a solid lesion and patients with a cystic lesion, based on the tumour type of the largest resected tumour as identified through preoperative imaging or during pathological examination. Patients having both solid and cystic PanNETs were allocated to the group corresponding to the type of the largest lesion. The histological grading of neuroendocrine tumours was conducted using the 2022 World Health Organization (WHO) classification. Patients without available pathological examination reports were excluded from the analysis.

The study protocol was approved by the Medical Ethical Committees (METC) of the University Medical Centres in the Netherlands.

### Statistical analysis

Clinical characteristics are reported as mean values with standard deviations or medians with ranges, depending on the distribution of the data. Analyses were performed using IBM SPSS, version 27 (SPSS Inc., USA).

## Results

**Figure 1:**
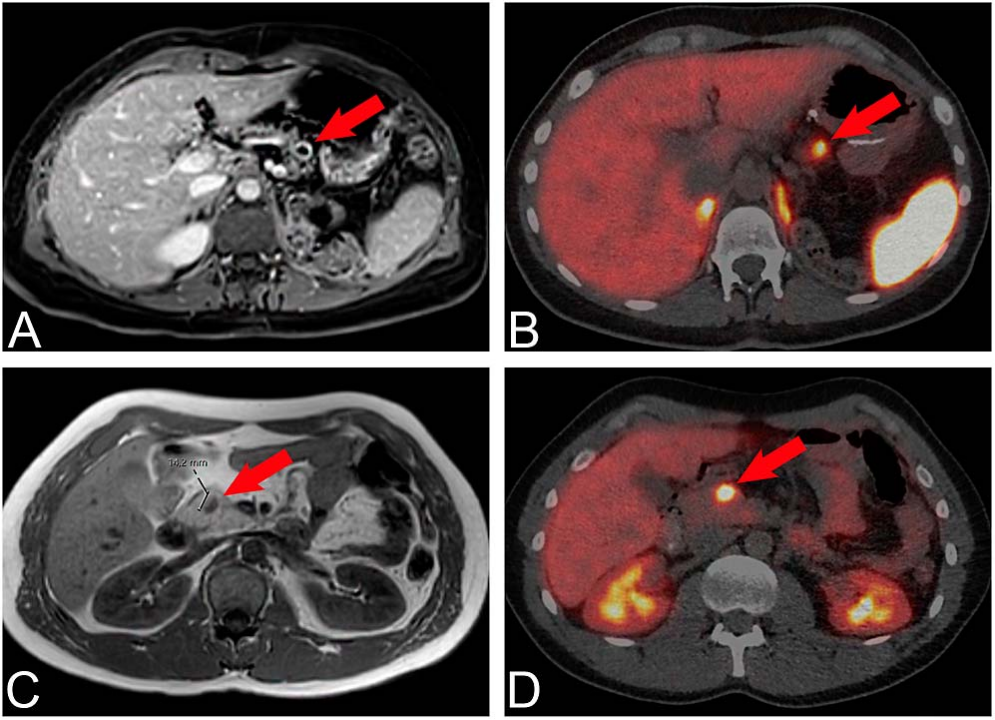
MRI scans and [^68^Ga]Ga-DOTATOC PET/CT scans of a cystic and solid pancreatic neuroendocrine tumour (PanNET). (A) MRI E-THRIVE image of a 14-mm cystic PanNET in the tail of the pancreas; (B) the corresponding [^68^Ga]Ga-DOTATOC PET/CT scan of the PanNET shown in A (SUVmax 13.1 and SUVmean 7.4). (C) MRI T1 image of a 14-mm solid PanNET in the head of the pancreas; (D) the corresponding [^68^Ga]Ga-DOTATOC PET/CT scan of the PanNET shown in C (SUVmax 13.7 and SUVmean 7.8).

A total of 136 patients with pancreatic lesions out of a total of 209 MEN1 patients were eligible for inclusion, comprising 60 (44%) men and 76 (56%) women. Patient characteristics are detailed in Table 1. Of the 136 patients with PanNETs, 33 patients (24%) exclusively had cystic PanNETs and 29 patients (21%) had both cystic and solid PanNETs. Cystic PanNETs were predominantly localised to the tail of the pancreas. Details are shown in Table 1. In addition to MRI or CT, a [^68^Ga]Ga-DOTATOC PET/CT scan was performed in 13 patients with both solid and cystic lesions, 34 patients with solid PanNETs and 13 patients with cystic lesions. In all patients, regardless of whether the lesions were solid or cystic, the lesions demonstrated [^68^Ga]Ga-DOTATOC PET/CT uptake. However, in some of the patients with multiple pancreatic lesions, only the largest lesions demonstrated activity. Figure 1 presents images of both an MRI scan and a corresponding [^68^Ga]Ga-DOTATOC PET/CT scan from a patient with a cystic PanNET and a patient with a solid PanNET. Both lesions showed similar standardised uptake values (SUVs): the cystic PanNET had an SUVmax 13.1 and SUVmean of 7.4, and the solid PanNET had an SUVmax of 13.7 and SUVmean of 7.8. These SUVs are consistent with those reported in previous studies (Virgolini *et al.* 2016).

**Table 1:** Patient characteristics of the whole study population.

	*n* (%)			
	Total	Both solid and cystic PanNETs^a^	Solid PanNETs	Cystic PanNETs
	*n* = 136	*n* = 29	*n* = 74	*n* = 33
Gender				
Male	60 (44)	14 (48)	34 (46)	12 (36)
Female	76 (56)	15 (52)	40 (54)	21 (64)
Age at first PanNET diagnosis	40 (±16)	43 (±15)	40 (±16)	40 (±16)
*Mean (SD), y*				
Age at the start of the follow-up	45 (±15)	48 (±14)	44 (±16)	43 (±15)
*Mean (SD), y*				
Site of PanNET				
Head	23 (17)	1 (3)	17 (23)	5 (15)
Body	11 (8)	0 (0)	10 (14)	1 (3)
Tail	36 (26)	2 (7)	18 (24)	16 (48)
Head and body	8 (6)	1 (3)	4 (5)	3 (9)
Head and tail	26 (19)	13 (45)	10 (14)	3 (9)
Body and tail	16 (12)	5 (17)	9 (12)	2 (6)
Head, body and tail	16 (12)	7 (24)	6 (8)	3 (9)
DOTATOC performed	60 (44)	13 (45)	34 (46)	13 (39)
DOTATOC uptake				
Yes, in all of the lesions	57	11	34	12
Yes, in some of the lesions	3	2	0	1
No	0	0	0	0
Treatments during follow-up				
Surgery, *indication*	38 (28)	9 (31)	21 (28)	8 (24)
*Size PanNET ≥20 mm*	*20*	*5*	*10*	*5*
*Growth PanNET*	*3*	*1*	*2*	*0*
*Functional PanNET*	*8*	*2*	*5*	*1*
*Limited lymph node and/or liver metastasis*	*6*	*1*	*3*	*2*
*Ectopic PanNET^b^*	*1*	*0*	*1*	*0*
SSA, *indication*	16 (12)	4 (14)	11 (15)	1 (3)
*Metastatic PanNET*	*7*	*2*	*5*	*0*
*Growth PanNET*	*2*	*0*	*2*	*0*
*Functional PanNET*	*7*	*2*	*4*	*1*
PRRT, *indication*	5 (4)	2 (7)	3 (4)	0 (0)
*Metastatic PanNET*	*4*	*2*	*2*	*0*
*Functional PanNET*	*1*	*0*	*1*	*0*
MRgRT, *indication*	3 (2)	0 (0)	2 (3)	1 (3)
*Growth PanNET*	*3*	*0*	*2*	*1*
Sunitinib, *indication*	1 (1)	1 (3)	0 (0)	0 (0)
*Metastatic PanNET*	*1*	*1*	*0*	*0*
Everolimus, *indication*	2 (1)	1 (3)	1 (1)	0 (0)
*Metastatic PanNET*	*2*	*1*	*1*	*0*

Abbreviations: PanNETs, pancreatic neuroendocrine tumours; Y, years; SD, standard deviation; SSA, somatostatin analogue; PRRT, peptide receptor radionuclide therapy; MRgRT, magnetic resonance-guided radiotherapy.

^a^ Both solid and cystic PanNETs simultaneously present.

^b^ Presumed to be a lymph node metastasis.

Table 2 presents the growth data of PanNETs. Four patients were excluded from this analysis due to the initiation of SSA treatment or PRRT before the start of follow-up. In addition, thirteen patients were excluded because their PanNETs were insufficiently visible on imaging scans, scan reports were unavailable or imaging was performed only once before surgery, making growth assessment unfeasible. The median follow-up was 5.1 years (range 0.5–13.1 years). The median growth of cystic PanNETs was 0.3 mm/year (range −1.4 to 5.1 mm/year), while the median growth of solid PanNETs was 0.2 mm/year (range −1.4 to 8.2 mm/year) and the growth in the group with both solid and cystic lesions was 0.1 mm/year (range −0.5 to 4.3 mm/year).

**Table 2:** Growth of PanNETs.

	Total	Both solid and cystic PanNETs^a^	Solid PanNETs	Cystic PanNETs
	*n* = 119	*n* = 20	*n* = 67	*n* = 32
Size largest PanNET at the first FU	9 (1–37)	9 (4–16)	9 (3–22)	8 (1–37)
Median, mm (range)				
Size largest PanNET at the last FU	10 (3–40)	11 (6–26)	10 (3–36)	10 (3–40)
Median, mm (range)				
Duration of FU	5.1 (0.5–13.1)	5.6 (1.0–11.0)	4.9 (0.5–13.1)	5.0 (1.0–13.0)
Median, year (range)				
Interval conventional imaging	12.4 (5.7–26.9)	12.1 (6.9–14.7)	12.4 (5.7–24.9)	12.4 (6.9–26.9)
Median, months (range)				
Growth PanNETs	0.2 (−1.4 to 8.2)	0.1 (−0.5 to 4.3)	0.2 (−1.4 to 8.2)	0.3 (−1.4 to 5.1)
Median, mm/year (range)				
Number of PanNETs at the end FU	2 (1–9)	3 (2–8)	2 (1–9)	2 (1–5)
Median (range)				

Abbreviations: PanNETs, pancreatic neuroendocrine tumours; FU, follow-up; mm, millimetres.

^a^ Both solid and cystic PanNETs simultaneously present.

Among patients with solid lesions, eleven out of 74 (15%) developed metastases during follow-up. Metastatic disease occurred in three out of 33 (9%) patients with cystic lesions and three out of 29 (10%) patients with both cystic and solid lesions simultaneously present. In the latter group, the largest lesion was cystic in all three cases. These results are displayed in Table 3. The sample sizes of the groups were too small for statistical analysis.

**Table 3:** Incidence of metastasis during follow-up.

	*n* (%)			
	Total	Both solid and cystic PanNETs^a^^,^^b^	Solid PanNETs	Cystic PanNETs
	*n* = 17	*n* = 3	*n* = 11	*n* = 3
Gender				
Male	7 (41)	2 (67)	4 (36)	1 (33)
Female	10 (59)	1 (33)	7 (64)	2 (67)
Age at metastasis	48 (±13)	49 (±12)	49 (±14)	43 (±17)
Mean (SD), y				
Tumour size largest PanNET at metastasis	19 (±9)	28 (±13)	17 (±6)	16 (±11)
Mean (SD), mm				
Largest PanNET at metastasis				
<10 mm	2 (12)	0 (0)	1 (9)	1 (33)
10–19 mm	7 (41)	1 (33)	5 (45)	1 (33)
≥20 mm	8 (47)	2 (67)	5 (45)	1 (33)
Site of metastasis				
Lymph nodes	12 (71)	3 (100)	7 (63)	2 (67)
Liver	3 (18)	0 (0)	3 (27)	0 (0)
Lymph nodes and liver	2 (12)	0 (0)	1 (9)	1 (33)

Abbreviations: PanNETs, pancreatic neuroendocrine tumours; SD, standard deviation; mm, millimetres.

^a^ Both solid and cystic PanNETs simultaneously present.

^b^ At metastasis, first patient had a 40-mm cystic and 7-mm solid PanNET, the second patient had a 29-mm cystic and 20-mm solid PanNET, and the third had a 15-mm cystic and 14-mm solid PanNET.

Between January 2010 and December 2023, 38 patients underwent surgery. Table 4 presents the characteristics of the largest resected tumours. Pathological examination confirmed that the lesions suspected to be cystic PanNETs on imaging were indeed PanNETs, and not other types of cystic pancreatic lesions. Cystic lesions had a diameter of 26 mm (standard deviation ± 11 mm) at the last imaging scan before surgery, while solid PanNETs measured 18 mm (standard deviation ± 7 mm). The indications for surgical resection of lesions smaller than 20 mm were either growth of the PanNETs, the presence of a functional PanNET or as a curative treatment of a locoregional metastasized PanNET. The Ki-67 index and mitotic count were comparable between cystic and solid lesions. Details of patients treated with PRRT, everolimus and sunitinib are provided in Supplementary material 1 (see section on Supplementary materials given at the end of the article).

**Table 4:** Characteristics of the largest resected tumour.

	*n* (%)		
	Total	Solid PanNET	Cystic PanNET
	*n* = 38	*n* = 23	*n* = 15
Gender			
Male	16 (42)	8 (35)	8 (53)
Female	22 (58)	15 (65)	7 (47)
Age at surgery			
Mean (SD), y	43 (±15)	42 (±16)	45 (±14)
Type of surgery			
Total pancreatectomy	3 (8)	0 (0)	3 (20)
Whipple/PPPD	8 (21)	4 (17)	4 (27)
Whipple/PPPD and distal pancreatectomy	4 (11)	2 (9)	2 (13)
Distal pancreatectomy	17 (45)	13 (57)	4 (27)
Distal pancreatectomy and enucleation caput	2 (5)	1 (4)	1 (7)
Enucleation caput	3 (8)	3 (13)	0 (0)
Enucleation corpus/tail	1 (3)	0 (0)	1 (7)
Indication for surgery			
Rapid growth or size >20 mm	23 (61)	12 (52)	11 (73)
Hormone producing PanNET	8 (21)	7 (30)	1 (7)
*Insulinoma*	*5 (13)*	*5 (22)*	*0 (0)*
*Gastrinoma*	*2 (5)*	*1 (4)*	*1 (7)*
*GHRHoma*	*1 (3)*	*1 (4)*	*0 (0)*
Limited lymph node and/or liver metastasis	6 (16)	3 (13)	3 (20)
Ectopic PanNET	1 (3)	1 (2)	0 (0)
Radiologic size largest lesion			
Mean (SD), mm	21.2 (±9.3)	18.1 (±7.0)	25.9 (±10.6)
Pathologic size largest lesion			
Mean (SD), mm	19.7 (±9.4)	16.9 (±7.9)	24.0 (±10.0)
Ki-67			
<3%	24 (63)	15 (65)	9 (60)
3–20%	9 (24)	7 (30)	2 (13)
>20%	0 (0)	0 (0)	0 (0)
Unknown	5 (13)	1 (4)	4 (27)
Mitotic count			
<2 hpf	25 (66)	13 (57)	12 (80)
2–20 hpf	3 (8)	2 (9)	1 (7)
>20 hpf	0 (0)	0 (0)	0 (0)
Unknown	10 (26)	8 (35)	2 (13)
NET grade (WHO)			
1	27 (71)	17 (74)	10 (67)
2	9 (24)	6 (26)	3 (20)
3	0 (0)	0 (0)	0 (0)
Unknown	2 (5)	0 (0)	2 (13)

Abbreviations: PanNETs, pancreatic neuroendocrine tumours; y, years; PPPD, pylorus-preserving pancreaticoduodenectomy; mm, millimetres; GHRHoma, growth hormone-releasing hormone-producing tumour; SD, standard deviation; hpf, high-power field; WHO, World Health Organization.

## Discussion

Our study demonstrated that cystic lesions suspected to be PanNETs by experienced radiologists were indeed confirmed as PanNETs based on their [^68^Ga]Ga-DOTATOC PET/CT uptake and pathological examination following resection. The growth of PanNETs appeared comparable for both cystic and solid lesions, as did the Ki-67 index and mitotic count and the number of patients who developed metastases during follow-up. However, due to the small sample size, analyses between the different groups were not conducted. To our knowledge, no other studies have specifically investigated cystic PanNETs in MEN1.

Our study has several strengths. First, it is the first investigation specifically focusing on cystic PanNETs in MEN1. The routine screening in MEN1 facilitates early detection of PanNETs, followed by structured monitoring, which provides valuable insights into the natural history of PanNETs. Second, our inclusion period starting from January 2010 adds robustness to our study, as advancements in imaging quality, pathological examination standards and electronic medical record documentation over time may have resulted in more reliable data collection. The extended follow-up period in our study further strengthens its validity.

Several limitations should also be discussed. The first is the retrospective design of the study. However, given the low prevalence of MEN1, this retrospective approach was necessary to ensure the inclusion of sufficiently large study groups. Even so, the subgroups were still too small to permit robust comparison. Despite the retrospective nature, the dataset had minimal missing data, and only a few patients had to be excluded due to unavailable imaging reports.

Second, the included PanNETs were generally small and slowly growing lesions. Consequently, even minor measurement errors could have significantly impacted the growth analysis, which may explain part of the negative growth observed in some lesions.

The pathogenesis of cystic PanNETs remains unclear, and several hypotheses exist regarding the mechanisms of their formation. Some researchers propose that necrosis, resulting from infarction caused by decreased blood supply from the fibrous capsule, contributes to the development of cystic lesions. However, tumour necrosis is rarely observed in cystic PanNETs, indicating that this may not be the primary pathogenesis of cystic lesion occurrence (Bordeianou *et al.* 2008, Singhi *et al.* 2012). Others suggest that intralesional haemorrhage is responsible for cystic degeneration (Takeshita *et al.* 1994, Ahrendt *et al.* 2002). Nonetheless, current evidence for this hypothesis is limited, as a previous study indicated that haemorrhage only occurred in larger PanNETs (Singhi *et al.* 2012). Some researchers argue that cystic PanNETs represent an independent subgroup, considering the distinct clinicopathological features found in prior studies, which predominantly focused on sporadic PanNETs (Zhu *et al.* 2019). The cystic lesions were associated with a lower TNM stage and displayed more favourable pathological features, including less frequent vascular and perineural invasion and lower Ki-67 indices and mitotic counts. However, in our study focusing on MEN1, such differences were not observed.

Our finding that cystic lesions in MEN1 are indeed PanNETs has significant clinical implications. Unfortunately, our sample size was too small for robust subgroup analyses, but the various types of PanNETs appear to exhibit similar growth rates and comparable Ki-67 indices and mitotic counts. Therefore, these lesions should be monitored in the same manner as solid PanNETs. When there is uncertainty about the nature of a cystic lesion, a [^68^Ga]Ga-DOTATOC PET/CT scan should be considered for further clarification. A notable finding in our study is that the average size of cystic PanNETs at the time of resection was larger than that of solid PanNETs. However, our study demonstrates that cystic lesions behave similarly to solid ones, suggesting that prolonged watchful waiting is not advisable.

Currently, surgical resection is recommended for non-functioning PanNETs exceeding 2 cm in size or demonstrating progressive growth (Niederle *et al.* 2021, van Beek *et al.* 2023). This cut-off size is based on the high risk of metastasis associated with lesions larger than 2 cm, which outweighs the morbidity associated with surgical complications. However, the studies that established this cut-off size did not perform subgroup analyses to assess differences in the risk of metastasis between solid and cystic PanNETs. Consequently, it remains unclear whether a different cut-off point might be appropriate for these two types of lesions. Further research is needed to address this gap. Studies exploring less invasive treatments than surgery for PanNETs are also important for improving clinical management. Examples of emerging local therapies include (magnetic resonance-guided) radiotherapy and radiofrequency ablation (van Vliembergen *et al.* 2023, Khoury *et al.* 2024). As explored in our study, solid and cystic lesions should be managed similarly, and therefore, these local treatment methods should be considered. When successful, such less invasive treatments might provide a more proportionate option for management of early-stage PanNETs, balancing the relative low risk of metastasis versus the morbidity of the treatment.

## Conclusion

This is the first study giving an overview of the natural behaviour of cystic PanNETs in MEN1, demonstrating [^68^Ga]Ga-DOTATOC PET/CT uptake in cystic PanNETs and a comparable natural course and findings at pathological examination.

## Supplementary materials



## Declaration of interest

The authors declare that there is no conflict of interest that could be perceived as prejudicing the impartiality of the work reported.

## Funding

This work was supported by KWFhttps://doi.org/10.13039/501100004622 (Dutch Cancer Society), a non-profit organisation (project number 13575).

## Author contribution statement

ENMV designed the study, collected the data, performed statistical analysis, interpreted the data and drafted the manuscript. CRCP designed the study, critically reviewed the manuscript and approved the final version of the manuscript. ACV critically reviewed the manuscript and approved the final version of the manuscript. AJATB created the figure and critically reviewed the manuscript. GDV designed the study, interpreted the data, critically reviewed the manuscript and approved the final version of the manuscript. JML designed the study, supervised the data collection, performed statistical analysis, interpreted the data, critically reviewed the manuscript and approved the final version of the manuscript.
