# Supplementary material for: The natural course of cystic pancreatic neuroendocrine tumours in MEN1
Source: Endocr Oncol. 2025 Feb 4;5(1):e240050. doi: 10.1530/EO-24-0050 (PMC11825168; doi:10.1530/EO-24-0050)
Supplement: Supplementary file 1 [file supplementary_materials.pdf]

Supplement 1. Details of patients with metastatic disease, treated with PRRT, everolimus, and sunitinib

**Patient**	**Year of diagnosis metastatic disease**	**PanNET type at diagnosis metastatic disease**	**Size at diagnosis metastatic disease**	**Ki-67 index**	**PanNET type during FU**	**PRRT**	**Start sunitinib**	**Start everolimus**
1	2005	Unknown[a]	Unknown[a]	5-10%[b]	Both solid and cystic	2006, 2008, 2010	N.A.	2012
2	2007	Cystic	54 mm	Unknown[c]	Both solid and cystic	2009, 2015	2017	N.A.
3	2009	Solid	22 mm	Unknown[c]	Solid	2010	N.A.	N.A.
4	2011	Solid	10 mm	15%[b]	Solid	N.A.	N.A.	2013
5	2018	Solid	15 mm	1%[d]	Solid	2019	N.A.	N.A.

Abbreviations: PanNET, pancreatic neuroendocrine tumour; PRRT, peptide receptor radionuclide therapy; FU, follow-up; N.A., not applicable

[a] Imaging results at the time of metastasis not available.

[b] Pathological examination of liver metastasis.

[c] Pathological examination was performed, but the Ki-67 index was not assessed.

[d] Pathological examination of pancreas performed in 2020, one year after the start of PRRT.
